# Cutaneous reporting patterns of drugs relevant to hepatocellular carcinoma: a cross-indication FAERS analysis and proposed management framework

**DOI:** 10.3389/fmed.2026.1959163

**Published:** 2026-10-30

**Authors:** Xiubo Sun, Zhe Liu, Jing Zhang, Lijie Zhai, Ling Yu

**Affiliations:** 1 Department of Pharmacy, The Second Hospital of Jilin University, Changchun, Jilin, China; 2 Department of Dermatology, The Second Hospital of Jilin University, Changchun, Jilin, China

**Keywords:** cutaneous adverse events, disproportionality analysis, hepatocellular carcinoma, immune checkpoint inhibitor, pharmacovigilance, tyrosine kinase inhibitor

## Abstract

**Background:**

First-line therapy for unresectable hepatocellular carcinoma (HCC) includes combinations of an immune checkpoint inhibitor (ICI) with an anti-angiogenic agent or tyrosine kinase inhibitor. Cutaneous adverse events are frequent, but immune-mediated eruptions and drug-induced reactions such as hand–foot skin reaction are routinely counted together, distorting the apparent skin toxicity signal and risking inappropriate interruption of the checkpoint inhibitor.

**Methods:**

In a disproportionality analysis using cross-indication reports from the US FDA Adverse Event Reporting System via openFDA, we computed reporting odds ratios (RORs; signal: ≥3 reports and lower 95% confidence limit >1) for 16 cutaneous reaction search phrases across 9 ICIs and 5 anti-angiogenic/multikinase agents relevant to HCC or used as class comparators. Proportional reporting ratios and information components were sensitivity measures. We examined co-reporting within selected combinations; reports were not restricted to HCC. An independent quarterly data sensitivity analysis compared primary-suspect, primary/secondary-suspect, and any-role coding with and without case version consolidation.

**Results:**

In the original analysis, vitiligo (ROR up to 40.95 for ipilimumab) and lichen planus generated signals for the six more extensively represented ICIs but not for the five anti-angiogenic/multikinase agents. Hand–foot skin reaction signals emerged for all five anti-angiogenic/multikinase agents (ROR up to 135.40); durvalumab and tremelimumab did not generate ROR signals. Severe reactions (Stevens–Johnson syndrome, toxic epidermal necrolysis) generated signals for several ICIs and some multikinase agents. Of 91 ROR signals, 68 also met both sensitivity criteria. Hand-foot RORs decreased after excluding reports containing selected co-reported medicines (atezolizumab: from 4.19 to 1.82 after excluding bevacizumab-containing reports), suggesting possible confounding by concomitant therapy without establishing drug causality. Camrelizumab and sintilimab had zero reports in the original generic-name query output, and tislelizumab had limited coverage. Quarterly primary-suspect analyses retained all five anti-angiogenic/multikinase hand–foot signals, with agent/event-specific exceptions among ICIs.

**Conclusions:**

These cross-indication reporting patterns focus attention on concomitant medicines but do not establish HCC-specific risks or individual drug causality. A proposed multidisciplinary monitoring framework with pharmacist involvement combines these observations with external clinical evidence on reactive cutaneous capillary endothelial proliferation, systemic organ assessment, and severe reactions. The framework and monitoring schedule have not been prospectively validated.

## 1 Introduction

Systemic therapy for unresectable hepatocellular carcinoma (HCC) has moved from single-agent tyrosine kinase inhibitors (TKIs) to combinations built on immune checkpoint inhibition. The IMbrave150 trial established atezolizumab plus bevacizumab over sorafenib for overall and progression-free survival (1), and further first-line options have followed, including sintilimab plus a bevacizumab biosimilar (ORIENT-32), camrelizumab plus rivoceranib (CARES-310), single-agent tislelizumab (RATIONALE-301), and the dual-immune checkpoint regimen durvalumab plus tremelimumab (HIMALAYA). We refer to the immune checkpoint inhibitor (ICI)-plus-anti-angiogenic/TKI combinations collectively as “targeted immunotherapy.”

The skin is among the earliest and most frequently affected organ systems under immune checkpoint inhibition. Cutaneous immune-related adverse events (cirAE) occur in a substantial proportion of ICI-treated patients: 18.1% in a multi-institutional cohort (2) and 27.5% in an atezolizumab meta-analysis (3), rising to approximately 34% with regimens targeting programmed cell death protein 1 (PD-1) or programmed death-ligand 1 (PD-L1), and 43%-45% with regimens targeting cytotoxic T-lymphocyte-associated protein 4 (CTLA-4) (4). A minority of ICI-treated patients progress to severe cutaneous adverse reactions (SCAR), such as Stevens–Johnson syndrome/toxic epidermal necrolysis (SJS/TEN), drug reaction with eosinophilia and systemic symptoms (DRESS) (5).

In HCC, the evaluation is more complex than under ICI monotherapy, because the two drug classes in a regimen produce different kinds of skin toxicity. The anti-angiogenic agents and multikinase inhibitors carry their own cutaneous profile (hand–foot skin reaction, acneiform eruption, and cholestatic pruritus), while camrelizumab produces a characteristic reactive cutaneous capillary endothelial proliferation (RCCEP) that is easily mistaken for an inflammatory lesion (6). The clinical cost of not separating these is visible in the incidence literature itself: Strictly defined rash under atezolizumab–bevacizumab has been reported in as few as ∼1.3% of HCC patients (7), whereas broader definitions that count all “skin reactions” reach substantially higher figures. Dose and interval were associated with acute cutaneous phenotypes in a small retrospective series (8). If immune-mediated and drug-induced events are pooled, apparent skin toxicity rates and any risk model built on them are distorted, and management can wrongly interrupt the ICI for a reaction caused by the partner agent.

Prior cirAE reviews have been either pan-cancer and mechanism-focused (4) or have addressed ICI toxicity in HCC across all organ systems without a cutaneous focus (9). A recent US FDA Adverse Event Reporting System (FAERS) study characterised cutaneous toxicities across ICIs in a general manner (10). We compared cutaneous reporting patterns for agents relevant to HCC and class comparators across all reported indications and examined how estimates changed after excluding a co-reported partner. HCC provides the clinical application context, not an eligibility restriction. We combined the descriptive findings with external clinical evidence to propose a multidisciplinary monitoring framework with pharmacist involvement (summarised in Figure 1).

**Figure 1:**
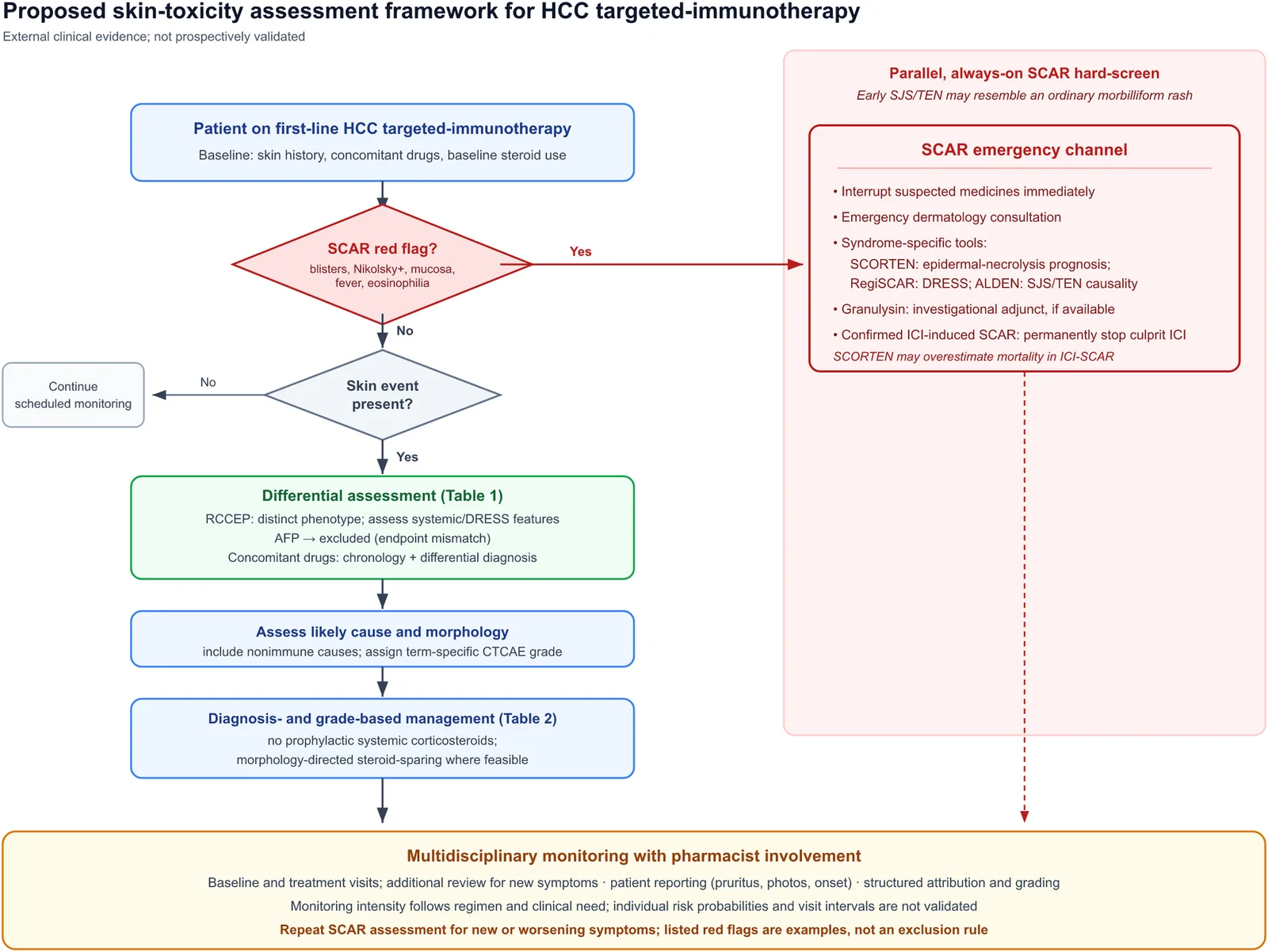
Proposed, not prospectively validated, confounder-aware clinical assessment framework for cirAE during first-line targeted immunotherapy for HCC. The main pathway proceeds from baseline assessment through confounder separation (Table 1), confirmation and grading, and CTCAE-based graded management (Table 2). An independent, always-on SCAR hard screen runs in parallel and, once triggered, escalates regardless of any low-grade stratification. The clinical actions and proposed multidisciplinary monitoring process with pharmacist involvement derive from external evidence; red flags are examples, and an initially negative assessment does not exclude later SCAR progression. ALT, alanine aminotransferase; AST, aspartate aminotransferase; AFP, alpha-fetoprotein; TKI, tyrosine kinase inhibitor; RCCEP, reactive cutaneous capillary endothelial proliferation; SJS/TEN, Stevens–Johnson syndrome/toxic epidermal necrolysis; DRESS, drug reaction with eosinophilia and systemic symptoms; CTCAE, common terminology criteria for adverse events.

**Table 1:** Proposed assessment domains in the HCC-targeted immunotherapy setting.

Confounder	What it actually reflects	Why it must be separated	Action
Camrelizumab-associated RCCEP	Reactive cutaneous vascular proliferation with proposed immune–angiogenic mechanisms (6, 15)	Distinct phenotype and natural history; treatment evidence remains limited	Dedicated channel: identify, grade, and manage independently; not in inflammatory scoring
ALT/AST elevation	Hepatic involvement (liver disease, tumour progression, drug hepatotoxicity, immune hepatitis)	Not a measure of skin extent; may nevertheless indicate systemic involvement in DRESS	Assess liver disease in parallel; retain hepatic/other organ assessment when SCAR or DRESS is suspected (16, 17)
Alpha-fetoprotein (AFP)	Tumour burden and treatment response marker	Endpoint mismatch with cutaneous outcomes	Exclude; use skin endpoints (type, extent, grade, pruritus, quality of life)
Possible concomitant anti-angiogenic/TKI skin toxicity	Co-reported hand–foot skin reaction and acneiform dermatitis (this study)	May influence reported ICI associations; co-reporting alone cannot establish causality (Section 3.5)	Review chronology and alternative causes; use ALDEN only for SJS/TEN-specific assessment (18)

Clinical actions draw on external evidence and have not been validated by this FAERS analysis.

**Table 2:** Proposed summary of external CTCAE-based management guidance, with a parallel SCAR pathway; not a validated protocol.

CTCAE grade	ICI	Skin-directed treatment	Systemic corticosteroid	Referral/setting
G1 (mild)	Continue	Topical corticosteroid, emollient, oral antihistamine	No	Intensified monitoring, patient education
G2 (moderate)	Continue or hold suspected agent per type/extent	Potent topical corticosteroid; systemic treatment when indicated	Dose and taper by diagnosis and response	Dermatology assessment
G3 (severe)	Hold	Supportive; morphology-directed steroid-sparing where feasible (28–30)	Systemic at guideline dose	Dermatology–oncology co-management; second-line immunomodulation if refractory
Confirmed ICI-induced SCAR/life-threatening ICI skin toxicity	Permanently discontinue culprit ICI	SCAR pathway	Per SCAR pathway	Hospitalisation, multidisciplinary care
**SCAR red flag (any grade)**	**Stop suspected drug immediately**	**Emergency dermatology consultation**	**Per SCAR pathway**	**Independent hard screen—escalate regardless of stratification**

This proposed summary is based on the cited external guidelines. Grading is specific to the CTCAE term; not every skin term has a grade 4 category. Suspected SCAR requires urgent interruption and evaluation, whereas confirmed ICI-induced SJS/TEN or DRESS warrants permanent discontinuation of the culprit ICI. Other suspect medicines do not settle ICI causality. Do not deliberately re-expose a patient to a suspected SCAR culprit. Systemic corticosteroids are used when clinically indicated, rather than routinely for prophylaxis.

Bold text in the final row highlights the SCAR red-flag pathway, which requires urgent action at any CTCAE grade, irrespective of routine severity stratification. CTCAE, common terminology criteria for adverse events.

## 2 Methods

### 2.1 Data source

We queried the FAERS through the openFDA drug event application programming interface (API; https://open.fda.gov/), which exposes de-identified, publicly available spontaneous reports. FAERS reports are unvalidated and reflect reporting rather than incidence; the disproportionality methods mentioned in the following are designed for this data type. Data reflect the database as accessed in July 2026. The original queries covered all reporting dates available through openFDA and imposed no HCC indication, age, country, or receipt date restriction. The exact historical extraction day and response date metadata were not retained; July 2026 denotes access rather than a patient observation period. openFDA retains the latest available version for each safety report identifier (11). We relied on that API-level handling and did not independently link different identifiers that might describe the same clinical case. The original aggregate tables contain no identifiers for retrospective case matching.

### 2.2 Agents and cutaneous events

We studied nine immune checkpoint inhibitors relevant to HCC or used as class comparators—atezolizumab, durvalumab (anti-PD-L1); pembrolizumab, nivolumab, camrelizumab, sintilimab, tislelizumab [anti-PD-1]; and ipilimumab and tremelimumab (anti-CTLA-4)—and five anti-angiogenic/multikinase agents—bevacizumab (anti-VEGF), lenvatinib, sorafenib, regorafenib, and cabozantinib. The original queries used 16 pre-specified reaction search phrases in the MedDRA-coded reaction field, grouped for presentation as immune pattern (rash, rash maculo-papular, pruritus, lichen planus, psoriasis, vitiligo, bullous pemphigoid, dermatitis bullous, SJS, TEN, DRESS, and erythema multiforme) or TKI/anti-angiogenic pattern [palmar-plantar erythrodysaesthesia syndrome (hand–foot skin reaction), dermatitis acneiform, alopecia, and skin fissures]. The scripts used quoted phrases in patient.reaction.reactionmeddrapt, not the full-term .exact field; longer reaction terms can therefore match a phrase. These groupings do not adjudicate biological mechanism. The original “bullous pemphigoid” query output contained only zeros; a separate *post hoc* query of the recorded term “pemphigoid” is reported in the supplementary analysis.

### 2.3 Disproportionality analysis

Reports were counted where an agent appeared in any role (suspect or concomitant medication). For each agent–event pair, we built a 2 × 2 table of report counts (agent and event, *a*; agent total; event total; and the total number of reports containing at least one MedDRA reaction term) and computed the reporting odds ratio, ROR = (a · d)/(b · c), with a 95% confidence interval from the standard error of the log-ROR. A Haldane–Anscombe 0.5 correction was added to all four cells when at least one cell was zero and all table margins were positive. Zero-margin comparisons are reported as not estimable in the corrected historical and revision tables; their counts are retained. A signal was defined conservatively as at least three reports and a lower 95% confidence limit above one; no adjustment was made for multiple comparisons. As sensitivity analyses, we recomputed, from the same counts, the proportional reporting ratio (PRR; signalling at PRR ≥ 2, *χ*^2^ ≥ 4, and ≥ 3 reports) and the information component [IC, with the approximate lower limit of the two-sided 95% credibility interval, IC025, using the formula described by Norén et al. (12); signalling at IC025 > 0], and regarded a signal as robust when it was concordant across all three metrics. Analyses used Python 3.12 with system curl for HTTPS requests. The analysis and sensitivity scripts and the full result tables are provided as supplementary reproducibility files. Reports may contribute to several agent–event combinations; therefore, the 224 combinations and summed drug counts do not reflect unique patient counts. The historical background comprised 20,328,571 reports. To ensure reproducibility, we performed an extraction on 10 September 2026 using a common first-FDA-receipt interval from 1 January 2004 to 30 June 2026, which included the current latest report versions and a reaction requirement for every count. Original phrase and complete-term queries were compared separately. Full counts, query definitions, error handling, and computational checks are documented in the Supplementary Material. The study is reported as a hypothesis-generating signal detection analysis, conducted in accordance with the Reporting of a Disproportionality Analysis for Drug Safety Signal Detection Using Individual Case Safety Reports in Pharmacovigilance (READUS-PV) guidelines for disproportionality analyses (13); a completed READUS-PV checklist is provided in the Supplementary Material.

### 2.4 Co-reporting and partner exclusion

For atezolizumab–bevacizumab, nivolumab–cabozantinib, and pembrolizumab–lenvatinib, the co-reporting proportion was calculated as partner-containing reports divided by all index-medicine/event reports. The original script examined 14 specified event terms, listed in the Supplementary Material. Partner exclusion removed every report containing the selected partner from the entire analysis universe; index-drug totals, event totals, and all four table cells were then recalculated. This changes the reference background and the index group, does not remove all possible confounding, and does not define monotherapy.

### 2.5 Management–framework synthesis

To complement the empirical analysis, we synthesised primary clinical studies, multi-omics and mechanistic reports, methodological standards for clinical prediction models, and current international and Chinese management guidelines, with an emphasis on first-line HCC-targeted immunotherapy. Where evidence is extrapolated from other tumour types or from ICI monotherapy, this is stated as such. This constitutes a focused narrative synthesis of external evidence; the proposed framework, its monitoring schedule, and its clinical performance were not tested in FAERS and have not been prospectively validated.

### 2.6 Drug role and case version sensitivity

We independently reconstructed 62 quarterly AERS/FAERS ASCII extracts [2011Q1–2026Q2 (14)]. After applying available deleted case lists and consolidating repeated snapshots of the same report identifier, we compared distinct report versions with one selected version per CASEID (legacy AERS: CASE). Case selection retained the latest FDA_DT, breaking ties by the highest numeric PRIMARYID/ISR and then source quarter. Eligibility required the selected version's FDA_DT to fall between 1 January 2011 and 30 June 2026 and a non-empty reaction; if the latest version was ineligible, it was not replaced by an older version. This population differs from the openFDA extraction in terms of source coverage, receipt date field, and drug name processing.

Drug identity and role were matched within the same drug row. A fixed generic/trade name dictionary was applied to non-empty PROD_AI, or to DRUGNAME when PROD_AI was absent. Any-role, primary-or-secondary-suspect (PS + SS), and primary-suspect-only (PS) definitions were compared using the same eligible background within each version policy. Each report contributed once per agent and event; an index-agent concomitant report could enter the complement under PS-only coding. The 16 original strings were matched as complete reaction terms, with PEMPHIGOID additionally evaluated as a separately labelled terminology correction. All 1,428 comparisons are supplied. Reader-facing quarterly tables report RORs and intervals only for joint counts ≥3; complete counts and arithmetic diagnostics are retained separately. Section S6 of the Supplementary Methods (Data Sheet 4) details source coverage, selection rules, and checks.

## 3 Results

### 3.1 Reporting coverage

Report volumes varied widely across agents: pembrolizumab (100,432 reports), nivolumab (94,833), bevacizumab (126,265), ipilimumab (42,014), atezolizumab (37,433), and cabozantinib (46,986) were well represented, whereas the original harmonised generic-name query output contained zero reports for camrelizumab and sintilimab and only 133 for tislelizumab. Zero-query output does not establish the absence of clinical adverse events or reports under other fields; the new extraction distinguished confirmed no matches from request errors. The original generic-name analysis therefore cannot characterise cutaneous reporting for some agents used in Chinese HCC practice, an important limitation that itself argues for locally sourced data (Section 4).

### 3.2 Immune pattern reporting among evaluable checkpoint inhibitors

In the original analysis, vitiligo and lichen planus generated signals for the six more extensively represented ICIs but not for the five anti-angiogenic/multikinase agents (Figure 2, Table 3). All-role drug mentions do not establish monotherapy. Vitiligo showed the strongest and cleanest separation: reporting odds ratios of 40.95 (95% CI, 35.06–47.84) for ipilimumab, 27.25 (23.90–31.08) for nivolumab, 21.74 for pembrolizumab, 14.03 for tremelimumab, 9.70 for atezolizumab, and 6.87 for durvalumab (all six of these ICIs met the signal criterion), against no signal for any anti-angiogenic/multikinase agent (ROR 0.14–1.56). Lichen planus also generated signals for these six ICIs (ROR 4.83–12.73) and for none of the five anti-angiogenic/multikinase agents. This pattern is consistent with the known ipilimumab–vitiligo association, but does not independently validate other reporting signals. The vitiligo signal may be influenced by the melanoma indication, in which on-treatment vitiligo is well recognised; these estimates describe cross-indication reporting rather than HCC-specific behaviour.

**Figure 2:**
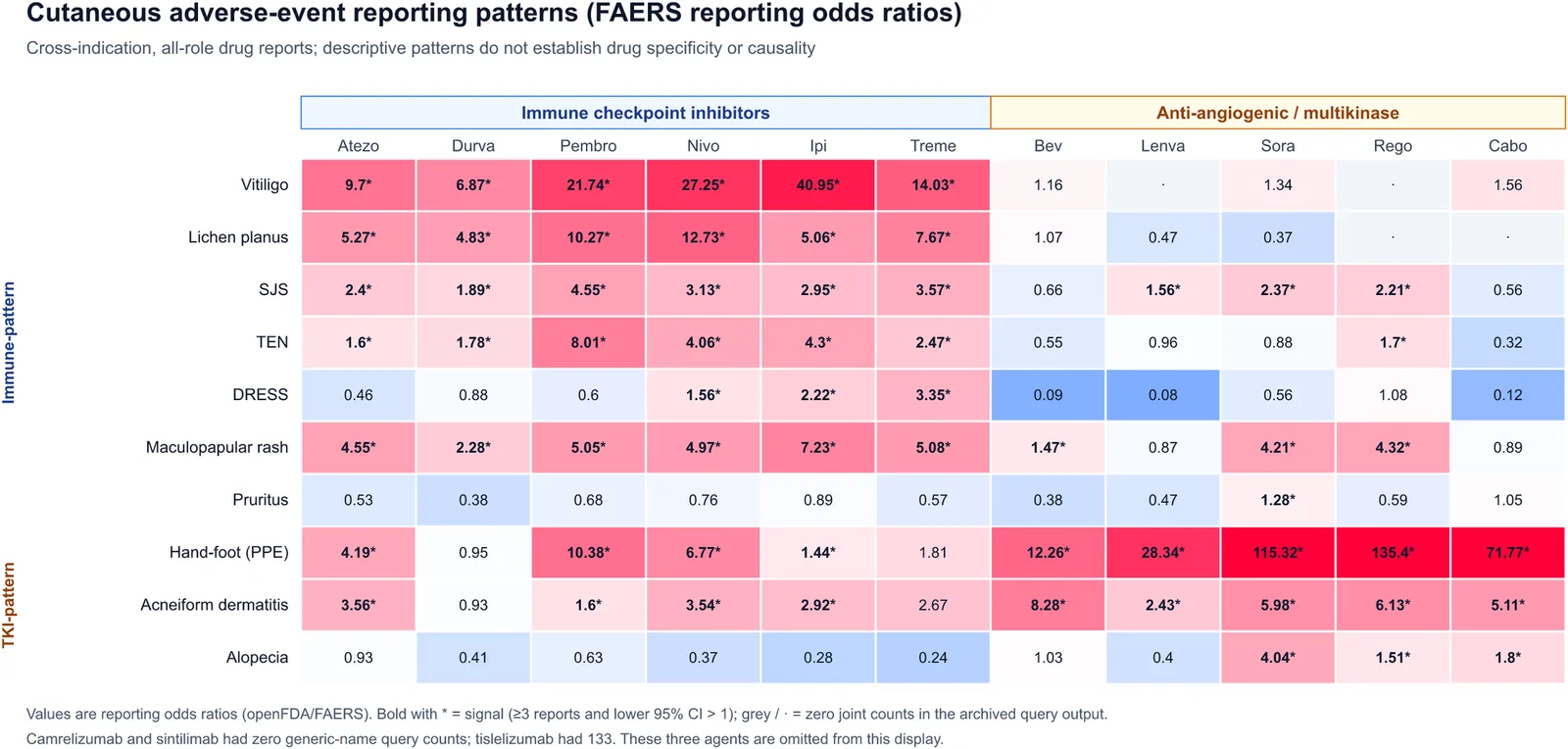
Cross-indication cutaneous reporting patterns across immune checkpoint inhibitors and anti-angiogenic/multikinase agents (original July 2026 FAERS RORs, quoted-phrase reaction searches). Bold with an asterisk denotes a signal (≥3 reports and lower 95% CI above 1); grey cells denote zero counts in the archived query output, not absence of clinical risk. The original display omits camrelizumab and sintilimab (zero generic-name query counts) and tislelizumab (133 reports); counts and estimability information for all 14 agents remain in the supplementary tables. The plot does not establish drug specificity or causality.

**Table 3:** Selected cross-indication cutaneous reporting estimates (original FAERS RORs and 95% CIs).

Cutaneous query	Representative ICI estimate	Representative anti-angiogenic/TKI estimate	Descriptive pattern
Vitiligo	Ipilimumab 40.95 (35.06–47.84); nivolumab 27.25 (23.90–31.08)	Bevacizumab 1.16 (0.71–1.90); sorafenib 1.34 (0.43–4.14) (no signal)	Stronger ICI reporting
Lichen planus	Nivolumab 12.73 (10.81–15.00); pembrolizumab 10.27 (8.62–12.24)	Sorafenib 0.37 (0.05–2.59); bevacizumab 1.07 (0.67–1.71) (no signal)	Stronger ICI reporting
SJS/TEN	Pembrolizumab TEN 8.01 (7.36–8.72); SJS 4.55 (4.15–4.98)	Sorafenib SJS 2.37 (1.80–3.11)	Signals across classes
Hand–foot skin reaction	Durvalumab 0.95 (0.61–1.47); tremelimumab 1.81 (0.90–3.62) (no signal)	Regorafenib 135.40 (127.90–143.33); sorafenib 115.32 (110.08–120.82)	Stronger anti-angiogenic/TKI reporting
Acneiform dermatitis	Pembrolizumab 1.60 (1.20–2.14)	Bevacizumab 8.28 (7.35–9.33); regorafenib 6.13 (3.95–9.51)	Stronger anti-angiogenic/TKI reporting

These are all-role, query-defined report comparisons, not treatment incidence or causal contrasts. A non-signal does not establish absence of risk. The pembrolizumab–acneiform dermatitis estimate meets the ROR criterion but not all three signal criteria.

### 3.3 Hand–foot skin reaction reporting

In the original analysis, a different reporting pattern was observed for hand–foot skin reaction. Hand–foot skin reaction (palmar-plantar erythrodysaesthesia syndrome) generated signals for every anti-angiogenic/multikinase agent, with reporting odds ratios of 135.40 for regorafenib, 115.32 (110.08–120.82) for sorafenib, 71.77 for cabozantinib, 28.34 for lenvatinib, and 12.26 for bevacizumab, whereas durvalumab (0.95) and tremelimumab (1.81) did not meet the signal criterion; these estimates do not identify monotherapy exposure. Ipilimumab reached only a marginal reporting odds ratio of 1.44 (95% CI, 1.13–1.83), an order of magnitude below the anti-angiogenic range; this value did not meet the PRR threshold and so was not concordant across all three metrics. Acneiform dermatitis likewise displayed the strongest signal for the anti-angiogenic/multikinase agents (ROR up to 8.28 for bevacizumab).

### 3.4 Severe reactions are shared but ICI-predominant

SJS and TEN generated signals predominantly for ICIs (for example, TEN ROR 8.01 for pembrolizumab and 4.06 for nivolumab; SJS ROR 4.55 for pembrolizumab), with weaker signals for some multikinase agents, consistent with their potential to cause drug hypersensitivity. Severe reactions therefore cannot be attributed to the ICI by default and require culprit drug adjudication (Section 4).

### 3.5 Sensitivity of ICI signals to co-reported partners

The estimates were sensitive to co-reported medicines. For atezolizumab, approximately 75% of its 175 hand–foot reports also listed bevacizumab, and the ROR attenuated from 4.19 to 1.82 when bevacizumab-containing reports were excluded. For nivolumab, approximately 79% co-reported cabozantinib, and the ROR changed from 6.77 to 1.73. Both reduced estimates still met the original ROR signal definition. For pembrolizumab, approximately 37% of 1,111 hand–foot reports also listed lenvatinib, and the ROR changed from 10.38 to 7.75 after exclusion. Historical co-reporting percentages are reported to the precision supported by the saved table; not all integer co-counts and margins were retained. Lenvatinib-excluded reports can still contain other medicines. Partner exclusion also attenuated several anti-angiogenic-associated signals (hand–foot by up to 3.9-fold, hypertension up to 3.06-fold, and proteinuria up to 13-fold), whereas vitiligo and lichen planus estimates changed less (full/excluded ROR ratios 0.78–0.93 and 0.88–0.98, respectively). These differences suggest possible confounding by concomitant therapy, but changes in indication and reference background composition are alternative explanations. Neither attenuation nor a residual signal identifies the culprit drug or establishes the presence or absence of an ICI effect.

### 3.6 Robustness across disproportionality metrics

Several representative signals were concordant across all three metrics. For ipilimumab–vitiligo, the ROR, PRR, and IC025 were 40.95, 40.79, and 4.84, respectively; for sorafenib–hand–foot skin reaction, they were 115.32, 103.28, and 6.45. Both examples met the PRR (≥2, *χ*^2^ ≥ 4) and IC (IC025 > 0) criteria in addition to the ROR criterion. Table 3 also includes weaker or non-signalling estimates; its pembrolizumab–acneiform dermatitis ROR signal did not meet all three criteria. Of 91 ROR signals overall, 68 (75%) also met both PRR and IC criteria, the discordant minority being the weakest associations. Concordance analysis assesses sensitivity to the metric but does not eliminate biases shared by the same report counts. The revision extraction included 20,692,302 reports in the specified universe. Phrase and complete-term analyses yielded 90 and 93 ROR signals, respectively (69 and 70 concordant across all three metrics). One borderline signal differed from the historical phrase analysis, and three weak signals differed between the new query definitions. The principal descriptive patterns persisted, but query definitions were not interchangeable. A separate *post hoc* PEMPHIGOID query identified reports missed by the original phrase, including signals for several ICIs and lenvatinib. Complete differences are supplied in the Supplementary Material.

### 3.7 Drug role and case version sensitivity

The quarterly sensitivity analysis included 21,777,640 eligible distinct report versions and 18,523,126 reports after CASEID selection. All five anti-angiogenic/multikinase hand–foot signals persisted under PS-only coding after case selection (RORs 9.22–130.41). The ipilimumab hand–foot estimate changed from 71 any-role reports, ROR 1.51 (95% CI 1.19–1.90), to 14 PS reports, ROR 0.64 (0.38–1.08). Unlike the original openFDA result, quarterly tremelimumab hand–foot reporting met the any-role criterion after case selection (11 reports; ROR 2.17, 1.20–3.93), but only one PS report remained. Several ICI vitiligo and lichen planus signals persisted; durvalumab–vitiligo had only two PS reports, and tremelimumab had no PS vitiligo or lichen planus reports. These sparse results do not establish the absence of risk. Atezolizumab, pembrolizumab, and nivolumab retained PS hand–foot signals, so concomitant role inclusion alone does not explain all such reporting. Suspect assignment does not establish causality.

CASEID selection changed 18 of the 714 agent–event–role signal classifications, including sparse or borderline signals; all changes and the complete SJS/TEN/DRESS subset are supplied in Section S6 of the Supplementary Methods (Data Sheet 4) and the accompanying tables. Camrelizumab and sintilimab were present in the quarterly source data (649 and 1,079 selected any-role reports), although PS-only coverage remained sparse (2 and 4 reports). These findings qualify individual signals while retaining the prominent descriptive patterns.

## 4 Discussion

In the original analysis of agents relevant to HCC and class comparators, cross-indication reports showed different cutaneous reporting patterns: Vitiligo and lichen planus generated signals among the six more extensively represented ICIs, hand–foot skin reaction was more strongly reported for anti-angiogenic/multikinase agents, and severe reactions occurred across groups. Partner-exclusion analyses (Section 3.5) suggest possible co-medication confounding without establishing causality. The proposed assessment framework (Table 1) combines these descriptive findings with external clinical evidence. The reporting patterns observed in FAERS support reviewing concomitant medicines and considering severe reactions during clinical assessment. The specific attribution methods, RCCEP pathway, systemic organ assessment, and treatment/monitoring recommendations in the proposed framework are drawn from published clinical literature. Neither these clinical procedures nor their benefits were validated by this analysis.

### 4.1 RCCEP through a dedicated channel

Camrelizumab-associated RCCEP is a reactive cutaneous vascular proliferation, characterised by a distinct vascular phenotype with proposed immune–angiogenic mechanisms (6), and can be identified, graded, and managed on a distinct clinical pathway based on external evidence (15). Under camrelizumab monotherapy, RCCEP occurred in 145 of 217 patients (66.8%), almost entirely grade 1–2, and its incidence rate fell when apatinib was added, consistent with anti-angiogenic suppression of vascular proliferation (6, 15). RCCEP also carries prognostic weight in HCC: Patients who developed it had markedly longer progression-free (median 3.2 vs. 1.9 months) and overall survival (17.0 vs. 5.8 months) than those who did not (6). Like the cirAE–survival association noted in the following, this prognostic link is subject to guarantee time and immortal time bias and should not be read as causal. Neither the original generic-name query nor the limited quarterly suspect role coverage characterises RCCEP; its proposed clinical use draws on external evidence and requires further evaluation.

### 4.2 Transaminases and alpha-fetoprotein as non-cutaneous measures

Alanine aminotransferase and aspartate aminotransferase (ALT/AST) reflect hepatic involvement (underlying liver disease, tumour progression, drug hepatotoxicity, or immune hepatitis) and require appropriate assessment rather than serving as skin-specific measures (16, 17). Liver and other organ abnormalities must nevertheless remain part of the evaluation for systemic reactions, particularly DRESS. Alpha-fetoprotein (AFP) is a tumour burden marker and an endpoint mismatch for cutaneous outcomes.

### 4.3 Clinical assessment of concomitant medicines

Section 3.5 shows that reporting estimates can change after excluding partner-containing reports, without identifying a causal drug. Skin events during combination therapy require assessment of medication chronology, morphology, alternative diagnoses, and the course after clinically indicated withdrawal. ALDEN is a drug causality algorithm for SJS/TEN, not a general rash score or severity scale (18). Deliberate re-exposure to a suspected SCAR culprit should not be used to test causality.

### 4.4 An independent SCAR hard screen

Because SJS/TEN signals across drug classes (Section 3.4) and a minority of cases begin as an ordinary rash (5), every rash assessment should run in parallel with a red-flag hard screen (blisters, epidermal detachment, positive Nikolsky sign, mucosal involvement, fever, and eosinophilia) that escalates independently of any low-grade stratification (19). SCORTEN, ABCD-10, and Re-SCORTEN address epidermal-necrolysis mortality prognosis, whereas RegiSCAR assists DRESS case classification; these tools are not interchangeable. Selected granulysin testing can supplement clinical assessment, but SCORTEN may overestimate mortality in ICI-associated populations and should not be a sole determinant (5, 20–23).

### 4.5 Endotype-directed graded management

Management follows grading based on the Common Terminology Criteria for Adverse Events (CTCAE) (24), informed by guidelines from the American Society of Clinical Oncology (ASCO), European Society for Medical Oncology (ESMO), and Society for Immunotherapy of Cancer (SITC), together with recent Chinese and multidisciplinary guidelines (16, 17, 25–27) (Table 2). Prophylactic systemic corticosteroids are not used to prevent cirAE: Baseline steroids equivalent to ≥10 mg/day prednisone have been associated with poorer outcomes during PD-(L)1 treatment, first shown in non-small-cell lung cancer and confounded by the poor-prognosis indications for which they are given (31). Because morphology may suggest a targetable pathway [eczematous/T-helper-2, psoriasiform/interleukin-17A, lichenoid/interferon-γ; (32)], steroid-sparing options can limit systemic immunosuppression: dupilumab for eczematous and pruritic disease (29), interleukin-17A blockade for psoriasiform toxicity (28), and non-corticosteroid maintenance for lichenoid reactions (30). On rechallenge, the same-class irAE recurs in a substantial minority (33).

### 4.6 Risk prediction and the value of the cirAE signal

No validated HCC-specific cutaneous prediction model exists (4, 7). An adjacent thyroid dysfunction nomogram reported internal validation 6-, 12-, and 24-month areas under the curve of 0.749, 0.755, and 0.745, respectively, for a different endpoint and population (34). Independent validation of prior irAE biomarkers has frequently failed (35). A usable tool would require adequate sample size (36), transparent reporting (37), bias appraisal (38), and external validation with proper calibration (39). Until then, per-factor reasoning and independent SCAR assessment may support clinical review, but individual probabilities, validated risk tiers, and expected external discrimination cannot be inferred from the present analysis. The observed association between cirAE and survival requires cautious interpretation: In HCC, irAE occurrence pooled across organ systems, rather than cutaneous events specifically, is associated with better progression-free survival (pooled hazard ratio 0.66) and, for grade 1–2 events, longer overall survival (hazard ratio 0.50) (40), consistent with the pan-cancer cirAE–survival association (overall survival hazard ratio ∼0.61) concentrated in low-grade disease (41). This association is subject to immortal time and responder bias and must not be assumed to extend to lethal SCAR.

### 4.7 Multidisciplinary monitoring with pharmacist involvement

Within the proposed multidisciplinary framework, pharmacists can contribute medication-related tasks alongside dermatology and oncology assessment (42–44): concomitant medication reconciliation, syndrome-appropriate medication review, reviewing baseline systemic corticosteroid exposure and its clinical indication, support for endotype-directed drug selection, and rechallenge support. The proposed monitoring process includes baseline assessment, review at treatment visits, and additional follow-up by symptoms and regimen; cycle lengths vary, and delayed reactions require continued vigilance. Prospective capture can pair clinician grading with patient self-report using validated instruments (the Patient-Reported Outcomes version of the Common Terminology Criteria for Adverse Events [PRO-CTCAE], an itch numerical rating scale, and a dermatology quality-of-life measure such as the Dermatology Life Quality Index [DLQI] or Skindex).

### 4.8 Comparison with prior work and limitations

Unlike a general ICI cutaneous FAERS analysis (10) or a broad FAERS cutaneous-irAE survey (45), we compared cross-indication reporting patterns for medicines relevant to HCC and examined sensitivity to co-reported partners, motivating further clinical assessment rather than establishing causal separation. The management framework informed by external clinical literature is a proposed synthesis of current evidence and consensus rather than a prospectively validated protocol; a de-identified HCC registry is the natural vehicle for evaluating it. The study inherits the limitations of spontaneous reporting: Reports are unvalidated, lack a treated-patient or person-time denominator, and are subject to reporting, indication, notoriety, and co-reporting biases. The original analysis counted any-role drug mentions and relied on openFDA's latest-version handling. The quarterly sensitivity independently compared drug roles and consolidated versions within CASEID (Section 3.7), but different CASEIDs may still describe the same clinical episode. Its date field, source coverage, and non-exhaustive drug name dictionary differ from the openFDA extraction; it therefore tests sensitivity within a separately defined population. Reporter-assigned suspect status does not adjudicate drug causality. Original API response metadata were not retained, and the old scripts could convert request errors to zeros, so historical zero counts could not all be independently classified. The openFDA revision extraction distinguishes confirmed no matches from errors; report version sensitivity was assessed separately in the quarterly reconstruction. Query strings also limit event coverage, including the unmatched original “bullous pemphigoid” phrase; this does not imply the absence of pemphigoid reports. No correction was made for multiple comparisons across the 224 agent–event pairs. Disproportionality is hypothesis-generating, not causal or incidence-estimating, and signals require independent confirmation; the dual threshold does not control the false discovery rate. openFDA retrieves drugs through a harmonised generic-name field populated mainly for US-registered products, so China-approved agents (camrelizumab, sintilimab, and in part tislelizumab) are under-retrieved. The results therefore describe cross-indication reporting patterns rather than camrelizumab-based Chinese practice. This is precisely the gap that would be filled by a prospective, de-identified HCC skin toxicity registry analysed in accordance with the sample size, reporting, bias, and external validation standards mentioned earlier.

## 5 Conclusion

Cross-indication reports for agents relevant to HCC show different cutaneous reporting patterns, with individual estimates sensitive to co-reported medicines, drug role coding, and report version selection. The findings do not establish HCC-specific risks or individual culprit drugs. They motivate a proposed, externally informed multidisciplinary monitoring framework with pharmacist involvement for medication review, RCCEP recognition, systemic organ assessment, and an independent SCAR pathway. The framework and monitoring process require prospective evaluation.

## Data Availability

Publicly available datasets were analysed in this study. The data can be found here: openFDA drug-event API: https://open.fda.gov/apis/drug/event/; FDA quarterly AERS/FAERS source extracts: https://fis.fda.gov/extensions/FPD-QDE-FAERS/FPD-QDE-FAERS.html. Aggregate results and reproducibility code are included in the Supplementary Material. Revision analyses are separately labelled and do not reconstruct an identical historical snapshot.

## Supplementary material

Supplementary Table S1 Completed READUS-PV reporting checklist.

Data Sheet 1 Corrected historical FAERS reporting odds ratio results.

Data Sheet 2 Corrected historical disproportionality sensitivity results.

Data Sheet 3 Historical partner co-reporting summary.

Data Sheet 4 Supplementary Methods and reproducibility.

Data Sheet 5 Reproducibility code and analysis outputs.
