# Supplementary material for: Cutaneous reporting patterns of drugs relevant to hepatocellular carcinoma: a cross-indication FAERS analysis and proposed management framework
Source: Front Med (Lausanne). 2026 Oct 30;13:1959163. doi: 10.3389/fmed.2026.1959163 (PMC13759389; doi:10.3389/fmed.2026.1959163)
Supplement: Supplementary file 1 [file Datasheet4.pdf]

Supplementary Methods and reproducibility

Manuscript: *Cutaneous reporting patterns of drugs relevant to hepatocellular carcinoma: a cross-indication FAERS analysis and proposed management framework*

The July 2026 analysis remains the main analysis. The separately identified revision analyses address reproducibility, event-query definitions, partner exclusion, drug-role restrictions and CASEID-based version selection. The September 10 openFDA analyses (S2–S5) and September 12 quarterly-file analyses (S6) use separately defined sources and reporting universes; their counts do not replace the historical results. File paths refer to the accompanying **science_bundle** archive after extraction.

S1. Historical data, reporting unit and report-version handling

The original analysis queried the openFDA drug-event endpoint at `https://api.fda.gov/drug/event.json`, which provides publicly released FAERS records. An adverse-event report can list several medicines and several reactions; their co-occurrence does not identify a causal medicine–reaction link. The analysis is cross-indication: HCC is the clinical application context, not an eligibility restriction. There was no HCC indication, age, country, primary-suspect, monotherapy or receipt-date filter. Medicines were retrieved through quoted strings in `patient.drug.openfda.generic_name` and could occur in any reported role. FDA drug-event overview.

The historical access month was July 2026. The exact extraction day, API release metadata and original response bodies were not retained. The original scripts did not specify a reporting-date interval. Consequently, July denotes access and cannot be converted retrospectively into a precise patient observation period or an as-known-at-cutoff snapshot. The background total of **20,328,571 reports containing a reaction** was preserved in the secondary analysis scripts. Original drug marginal queries did not explicitly repeat the reaction-existence requirement; the revision applies the same eligibility predicate to every count.

The unchanged provenance files `historical/faers_ror_results.csv` and `historical/faers_ror_sensitivity.csv` each contain 224 agent–event comparisons. Their saved agent, event and joint totals permit arithmetic reconstruction of all original RORs and both confidence limits at the reported precision. They do not authenticate the historical API extraction: original error handling could treat a request error as a zero match, and the absent response metadata cannot now establish which historical zeros were confirmed no-match responses. The separate historical partner file, `historical/systematic_coreporting.csv`, contains 42 rounded summaries but not all partner-removed margins or confidence limits. New data have not been inserted into these files. The revised reader-facing counterparts are `corrected_historical/faers_ror_results_corrected.csv` and `corrected_historical/faers_ror_sensitivity_corrected.csv`; they retain all counts and positive-margin historical estimates but mark the 44 zero-margin comparisons as not estimable.

The API documents that it returns the latest available version of each report. Its `duplicate=1` flag indicates that earlier versions were submitted; this flag was not used to discard the current record. For the original analysis and September 10 aggregate API extraction, we relied on upstream version handling and did not independently match report identifiers. Those aggregate marginals cannot support retrospective case matching. The independent quarterly-file analysis in S6 subsequently selects one version within CASEID and compares results before and after that selection. Neither analysis establishes unique patients or identifies the same clinical episode recorded under different CASEIDs. FDA fields reference. FDA's public conversion code and indexing code provide supporting implementation documentation for case-number-based processing; they are not an independent audit of every live record.

S2. Revision extraction, eligibility dates and report flow

The revision extraction was performed on **10 September 2026**. Every successful analysis response reported **`meta.last_updated=2026-07-30`**. One common universe was applied to all margins and intersections:

```
receivedate:[20040101 TO 20260630]
AND _exists_:patient.reaction.reactionmeddrapt
```

The interval is inclusive, from **1 January 2004 through 30 June 2026**, and uses `receivedate`, the date the report was **first received by FDA**. This differs from `receiptdate`, which records receipt of the most recent information. The extraction uses current latest versions of reports meeting the first-receipt condition, so later report updates may be included. It does not reconstruct either the July database snapshot or information available exactly on 30 June. FDA date-field definitions.

Stage	Number of reports
Available in the queried release	20,692,690
Excluded: no recorded reaction	3
With a recorded reaction, before receipt-date restriction	20,692,687
Additionally excluded: did not meet the first-receipt interval, including missing qualifying dates	385
Common revision analysis universe	**20,692,302**
Non-overlapping union mentioning at least one of the 14 study agents, within that universe	**466,166**

The study-agent union was obtained by one report-level OR query and was not calculated by adding overlapping agent totals. Its complement supplies reports mentioning none of the study agents, whereas each individual ROR uses the relevant index-drug complement. The union is an aggregate count of distinct database reports, not a count of unique patients. The 224 primary and 42 partner comparisons are also not report counts. A corresponding non-overlapping study-agent union was not preserved for the historical extraction.

The release, exact query predicates, statuses and counts are retained in `data/count_projections.json`. Table-specific report counts and all four cells are in the final CSVs listed in S5.

S3. Medicines, event definitions and comparison panels

S3.1 Medicines and query modes

The 14 original generic-name strings were retained: atezolizumab, pembrolizumab, nivolumab, durvalumab, tremelimumab, ipilimumab, camrelizumab, sintilimab, tislelizumab, bevacizumab, lenvatinib, sorafenib, regorafenib and cabozantinib. They comprise nine ICIs and five anti-angiogenic/multikinase agents relevant to HCC or used as class comparators. The drug search remained:

```
patient.drug.openfda.generic_name:"<generic name>"
```

For each event string, two analyses were performed in the identical revision universe:

```
Original-phrase mode: patient.reaction.reactionmeddrapt:"<event string>"
Exact-PT mode:        patient.reaction.reactionmeddrapt.exact:"<event string>"
```

The original field allows a phrase to occur within a longer reaction term; `.exact` uses the non-tokenized complete-term field. FDA's query documentation and field mapping support this distinction. The generic-name definition was not broadened or switched between modes. The complete machine-readable panel is `data/analysis_specification.json`.

S3.2 Original 16 cutaneous search strings

These are literal query strings. The group labels organize presentation and do not adjudicate the mechanism of individual reports.

No.	Literal event string	Presentation group
1	rash	Immune-pattern
2	rash maculo-papular	Immune-pattern
3	pruritus	Immune-pattern
4	lichen planus	Immune-pattern
5	psoriasis	Immune-pattern
6	vitiligo	Immune-pattern
7	bullous pemphigoid	Immune-pattern
8	dermatitis bullous	Immune-pattern
9	stevens-johnson syndrome	Immune-pattern
10	toxic epidermal necrolysis	Immune-pattern
11	drug reaction with eosinophilia and systemic symptoms	Immune-pattern
12	erythema multiforme	Immune-pattern
13	palmar-plantar erythrodysaesthesia syndrome	TKI/anti-angiogenic-pattern
14	dermatitis acneiform	TKI/anti-angiogenic-pattern
15	alopecia	TKI/anti-angiogenic-pattern
16	skin fissures	TKI/anti-angiogenic-pattern

All 14×16=224 planned comparisons are retained in each mode, including zero, rare, negative and nonsignalling results. The two modes therefore produce 448 rows. The unmatched original

"bullous pemphigoid" string remains unchanged in both modes; its separate terminology correction is described in S5.3.

S3.3 Three partner comparisons and all 14 partner-panel strings

The index/partner pairs were **atezolizumab/bevacizumab**, **pembrolizumab/lenvatinib** and **nivolumab/cabozantinib**. The 14 partner-panel strings included selected noncutaneous events to examine the broader reporting consequences of partner exclusion; they are not additional endpoints in the original 16-string cutaneous panel.

No.	Literal event string	Descriptive group
1	palmar-plantar erythrodysaesthesia syndrome	TKI-associated
2	hypertension	TKI-associated
3	proteinuria	TKI-associated
4	dysphonia	TKI-associated
5	diarrhoea	TKI-associated
6	dermatitis acneiform	TKI-associated
7	mucosal inflammation	TKI-associated
8	vitiligo	ICI-associated
9	lichen planus	ICI-associated
10	immune-mediated hepatitis	ICI-associated
11	hypophysitis	ICI-associated
12	pneumonitis	ICI-associated
13	colitis	ICI-associated
14	rash	Neutral comparison

All 3×14=42 planned pairs are retained per query mode, producing 84 rows. No low-count or nonsignalling pair is silently omitted.

S4. Count validation, statistics and partner exclusion

S4.1 Error handling and frozen count projections

The September 10 extraction used **864 endpoint requests**: 16 initial feasibility probes, two additional terminology probes, 828 unique primary/partner analysis queries after caching duplicates, three repeated stability queries and 15 post-hoc PEMPHIGOID queries. The main response cache contains 843 distinct analysis-query responses; the three stability responses are retained separately. All required analysis requests succeeded or returned an explicit confirmed no-match response. One ancillary field-count request returned HTTP 403 `API_KEY_MISSING`; it is recorded in `data/probe_projections.json` and was not interpreted as zero or used in calculations.

Only HTTP 404 with error code `NOT_FOUND` and the message `No matches found!` was accepted as a confirmed zero. HTTP 200 required valid, nonnegative integer total metadata. Authentication/schema failures could not become zero counts. The extraction implementation allowed bounded retries for transient transport, rate-limit and server errors, respecting Retry-

After and a finite request ceiling; no retry was required in the complete analysis extraction. Responses with inconsistent release metadata or invalid/negative four-cell tables would stop the analysis. Three start/end aggregate checks agreed. API parameters and access limits were consulted.

The supplied `data/count_projections.json` contains **query/count projections rather than full raw responses**: exact query parameters, request time, HTTP status, confirmed-zero/success classification, release metadata, total and SHA-256 of the original HTTP response body. Individual example report objects returned by `limit=1` have been omitted. The original full response bodies are retained separately; their hashes identify them but do not permit those bodies to be reconstructed from this smaller package. No individual-level example data or report identifiers are supplied in the projections. Ancillary probes that lacked an original byte-level hash or exact timestamp do not have those fields fabricated.

S4.2 Four-cell tables, ROR and confidence limits

For each drug A and event E within reporting universe U:

	Event E present	Event E absent
Drug A present	a = N(A∩E∩U)	b = N(A∩U)−a
Drug A absent	c = N(E∩U)−a	d = N(U)−N(A∩U)−N(E∩U)+a

All cells must be nonnegative integers and sum to N(U). ROR=ad/(bc). For a valid table with a zero cell and nonzero row/column margins, 0.5 is added to all four cells. Zero drug/event margins or zero complementary margins are **not estimable**; a continuity correction is not used to interpret those absent groups. Each revision mode contains 44 such main rows. Both corrected historical CSVs and the historical display suppress the 44 zero-margin estimates. These comprise 14 original “bullous pemphigoid” rows with a zero event margin and 32 camrelizumab/sintilimab rows with a zero drug margin, with two overlapping rows. The 180 positive-margin rows retain every historical value. The original numerical outputs remain in `historical/` for provenance only.

Using corrected cells where required, the two-sided 95% interval is:

```
exp{log(ROR) ± 1.96 × sqrt(1/a + 1/b + 1/c + 1/d)}
```

A ROR signal requires uncorrected a≥3 and a lower 95% limit >1. Counts below three remain in the tables but do not qualify as signals. The intervals describe statistical precision under the reporting-table model; they do not include all systematic reporting/indication biases or control multiplicity.

S4.3 PRR, Yates correction and IC025

PRR=(a/N_drug)/[(N_event−a)/(N−N_drug)]. The original screening criteria were a≥3, PRR≥2 and Yates-corrected chi-square≥4:

```
chi-square = N × max(|ad−bc|−N/2, 0)^2 / [(a+b)(c+d)(a+c)(b+d)]
```

The historical script omitted truncation at zero before squaring. Correcting it affects three very small historical values—durvalumab/dermatitis acneiform, tremelimumab/dermatitis bullous and tislelizumab/alopecia—all already rounded to 0.0 and below the screening threshold. No historical PRR or three-metric signal classification changes. The corrected implementation agreed with SciPy's `chi2_contingency(correction=True)` in all nondegenerate tables checked. Official SciPy documentation and implementation.

With expected count E=N_drug×N_event/N, the shrinkage IC and approximate lower 95% credibility limit are:

```
IC = log2[(a+0.5)/(E+0.5)]
IC025 = IC - 3.3(a+0.5)^(-1/2) - 2(a+0.5)^(-3/2)
```

These expressions correspond to equations 12–13 of **Norén GN, Hopstadius J, Bate A. Statistical Methods in Medical Research. 2013;22(1):57–69**, published online in 2011, doi:10.1177/0962280211403604. Their original full text, Section 3.1 recommends exact Gamma quantiles when a=0: log2 of the 0.025 posterior quantile with shape a+0.5 and rate E+0.5. This was implemented as `gamma.ppf(0.025, a=0.5, scale=1/(E+0.5))` for zero observed counts with nonzero margins; zero-margin tables remain not estimable. The method and the initial approximation are explicitly identified in separate columns. This correction does not affect the a≥3 signal criterion. SciPy Gamma parameterization.

IC screening requires a≥3 and IC025>0. Three-metric concordance requires all the ROR, PRR and IC criteria. Concordance uses the same underlying counts and therefore does not remove their shared biases or constitute independent validation.

S4.4 Partner-excluded reporting universe

For index drug A and selected partner B, all B-containing reports were removed from the **entire** analysis universe, defining U_withoutB=U∩NOT B. The restricted universe total was directly queried and verified against N(U)−N(B∩U). A's total, the event total and all four cells were independently recomputed within U_withoutB. Exact co-reports N(A∩B∩E∩U) were directly queried and checked using:

```
N(A∩E∩U) = N(A∩E∩U_withoutB) + N(A∩B∩E∩U)
co-reporting proportion = N(A∩B∩E∩U) / N(A∩E∩U)
```

Every full and restricted table is supplied with ROR and both confidence limits. The full/restricted ROR ratio is descriptive. Removing B changes both the exposed-report subset and its comparator, does not imply monotherapy and does not remove other medicines or unmeasured indication differences. Attenuation cannot identify a causal drug. No independent predictive claim is made from the relationship between co-reporting proportions and these mathematically related ROR ratios.

S5. Sensitivity results, term correction and offline reproduction

S5.1 Complete-panel results and differences

Analysis	Planned main pairs	ROR signals	Three-metric signals
Historical July phrase analysis	224	91	68

Analysis	Planned main pairs	ROR signals	Three-metric signals
Revision phrase analysis	224	90	69
Revision exact-PT analysis	224	93	70

Only regorafenib/toxic epidermal necrolysis changed ROR signal classification from historical to revision phrase analysis: the observed count remained 14, while the ROR shifted from 1.70 to 1.6887 at the lower-confidence-bound threshold. Within the same revision universe, exact-PT matching added three weak ROR signals compared with phrase matching: atezolizumab/rash, ipilimumab/pruritus and cabozantinib/pruritus. Thus the query definitions were not interchangeable. The principal descriptive contrasts for vitiligo, lichen planus and hand–foot reporting persisted, but neither absence of a signal nor stronger reporting in one class establishes clinical specificity. Every pair's change is retained in `tables/historical_phrase_vs_revision_phrase_vs_exact.csv`.

The historical-to-revision comparison combines release and explicit date-eligibility differences. The two revision modes isolate the event-query definition difference within the same reporting universe. These are new sensitivity results, not a reconstruction of an identical July snapshot.

S5.2 Partner-exclusion results

The hand–foot results were identical between the two revision query modes:

Index; excluded partner	Exact co-reports/full event reports	Full ROR (95% CI)	Restricted ROR (95% CI)
Atezolizumab; bevacizumab	135/180 (75.0%)	4.22 (3.64–4.89)	1.87 (1.40–2.51)
Pembrolizumab; lenvatinib	435/1,150 (37.8%)	10.42 (9.82–11.06)	7.62 (7.08–8.22)
Nivolumab; cabozantinib	574/725 (79.2%)	6.96 (6.46–7.49)	1.75 (1.49–2.06)

All three restricted estimates still met the ROR signal criterion. The phrase partner panel contained 39 full-set signals, of which two no longer met the criterion after partner exclusion: atezolizumab/hypertension and nivolumab/dysphonia. The exact-PT panel contained 40 full-set signals, with those two plus atezolizumab/rash no longer meeting the criterion after exclusion. The complete 84-row file retains both estimates for every comparison, rather than only the attenuated or statistically significant results.

S5.3 Separate post-hoc PEMPHIGOID correction

The original "bullous pemphigoid" phrase matched no reports in either query mode. The separately checked exact term **PEMPHIGOID** matched **7,314 reports** within the same revision universe. This correction was prompted by the terminology check and was not part of the original 16-string panel.

Drug	Joint reports	ROR (95% CI)	ROR signal
Atezolizumab	82	6.07 (4.88–7.55)	Yes
Pembrolizumab	377	10.73 (9.67–11.90)	Yes
Nivolumab	724	23.58 (21.83–25.47)	Yes
Durvalumab	56	7.91 (6.08–10.29)	Yes

Drug	Joint reports	ROR (95% CI)	ROR signal
Tremelimumab	8	5.38 (2.69–10.77)	Yes
Ipilimumab	149	10.04 (8.53–11.81)	Yes
Camrelizumab	0	Not estimable: zero drug margin	No estimate
Sintilimab	0	Not estimable: zero drug margin	No estimate
Tislelizumab	0	7.97 (0.50–127.87), zero-cell corrected	No
Bevacizumab	34	0.75 (0.53–1.05)	No
Lenvatinib	30	2.61 (1.82–3.74)	Yes
Sorafenib	1	0.14 (0.02–0.97), below the 3-report rule	No
Regorafenib	1	0.24 (0.03–1.74), below the 3-report rule	No
Cabozantinib	21	1.24 (0.81–1.91)	No

All 16 original event strings were checked: the other 15 complete strings retrieved reports. Six strings had different overall event counts under phrase versus exact matching, and 48 of the 224 drug–event intersections differed. A separate check on September 12 confirmed an explicit no-match response for exact “bullous pemphigoid” and 7,314 reports for exact “Pemphigoid” in the same openFDA universe. The returned reaction text was “Pemphigoid”; this verifies the recorded database label, not a clinically adjudicated bullous-pemphigoid diagnosis.

All seven positive ROR signals also met the PRR and IC criteria. The lenvatinib signal precludes an ICI-exclusive interpretation. A zero observed count with a wide continuity-corrected interval is not evidence of an increased risk. The 14 rows in `tables/posthoc_pemphigoid_14_rows.csv` remain separately marked post hoc and are not added to the 448/84 main sensitivity totals. The original zero-match wording does not establish absence of pemphigoid from FAERS.

S5.4 Supplied files and reproducibility checks

The accompanying science_bundle provides:

- `historical/faers_ror_results.csv`, `historical/faers_ror_sensitivity.csv` and `historical/systematic_coreporting.csv`: unchanged historical aggregate outputs.
- `historical_code/faers_ror.py`, `historical_code/make_sensitivity.py`, `historical_code/systematic_coreporting.py` and `historical_code/coreporting_probe.py`: unchanged original scripts for provenance only. Their online error handling and snapshot limitations are documented in `historical_code/README.md`; they are not the recommended reproduction route.
- `corrected_historical/faers_ror_results_corrected.csv` and `corrected_historical/faers_ror_sensitivity_corrected.csv`: reader-facing historical results with all 44 zero-margin comparisons not estimable; positive-margin sensitivity metrics retain their historical computational definitions.
- `corrected_historical/terminology_audit_all_16_phrases.csv`, `corrected_historical/zero_margin_rows_audit.csv` and `corrected_historical/README.md`: complete term audit, affected comparisons and interpretation.

- `tables/historical_display_224_rows.csv`: historical presentation with zero-margin estimates marked not estimable.
- `tables/table3_all_estimates_with_95ci.csv`: all 18 historical estimates displayed in Table 3, with both confidence limits.
- `tables/main_all_448_rows_final.csv` and `tables/partner_all_84_rows_final.csv`: all final revision results and four-cell counts.
- `tables/posthoc_pemphigoid_14_rows.csv`: separate post-hoc correction.
- `tables/historical_phrase_vs_revision_phrase_vs_exact.csv`: complete comparison with the historical results.
- `data/count_projections.json`, `data/probe_projections.json` and `data/analysis_specification.json`: aggregate provenance and literal query definitions, with no individual example report bodies.
- `reproduce.py`, `requirements.txt`, `MANIFEST.json` and `OFFLINE_VERIFICATION.json`: portable offline computation, dependencies, checksums and verification results.

With **Python 3.12** and **SciPy 1.17.1** installed, the single command `python reproduce.py` runs without network access or an external project directory. It reads the frozen projections, recalculates the 448-, 84- and 14-row final tables, compares all fields with the supplied references, and checks all 224 historical RORs and both confidence limits at their saved precision. It also rechecks report-count partitions and the three stability queries. Generated files are written to `reproduced/`; supplied reference files are not overwritten.

The September 10 calculation package was copied into a fresh independent temporary directory and executed from outside the package directory in Python isolated mode with socket network calls disabled. All three final CSVs reproduced **byte for byte** under CPython 3.12.10/SciPy 1.17.1, and all 224 historical ROR/CI checks passed. This validates the supplied offline calculations and portability; it does not retrospectively authenticate the historical extraction, quantify residual cross-ID duplication or establish clinical validity of the proposed framework.

The reader-facing historical CSV corrections can be regenerated without network access using `python reproduce_corrected_historical.py`; outputs are written to `reproduced/corrected_historical/`. This presentation correction does not replace the historical extraction or insert the post-hoc PEMPHIGOID results into its original panel.

S6. Independent quarterly-file drug-role and CASEID sensitivity

S6.1 Source, time interval and analysis scope

On **12 September 2026**, we analysed the official FDA ASCII quarterly releases from **2011Q1 through 2026Q2**, comprising 62 quarters each of DEMO, DRUG and REAC. The sources, filenames, sizes and SHA-256 values of all 186 files are supplied in `role_dedup/raw_source_manifest.json`; the manifest includes the official archive URLs. The FDA quarterly-release index and the technical notes within the source archives define the fields. These files expose the primary-suspect/secondary-suspect distinction required for this analysis, whereas openFDA's drug-characterization field combines suspect roles.

This sensitivity uses the date of FDA receipt for the reported version, **FDA_DT**, from 1 January 2011 through 30 June 2026 inclusive. It does not use openFDA `receivedate`, first receipt, event onset, or a July access-date snapshot. Its quarterly releases, date interval, ingredient/name ascertainment and complete-term event matching differ from the historical openFDA analysis. Consequently, within-quarterly comparisons assess role and version-selection sensitivity; differences between quarterly and historical estimates cannot be attributed to drug role or deduplication alone. The quarterly analysis remains cross-indication and is not an HCC-restricted cohort.

All 14 agents and the 16 original literal event strings were retained. PEMPHIGOID was additionally retained as the separately labelled terminology correction. Two version policies and three role definitions therefore generate **2×3×14×17=1,428 comparisons**, without selecting pairs by their results. The specification is in `role_dedup/protocol_locked.json` and the complete definitions are in `drug_dictionary.csv` and `event_dictionary.csv` in that directory.

S6.2 Drug identification, same-row roles and event counts

Drug identity uses nonempty PROD_AI; otherwise DRUGNAME is used. Text is lowercased, punctuation is normalized to spaces, and complete words are matched against the fixed generic/trade-name dictionary. Ingredient suffixes can therefore match their generic ingredient. This is a defined ascertainment rule rather than an exhaustive synonym dictionary or a replication of the openFDA harmonized generic-name field. An outcome- and role-blind name-coverage check found that all 22 Avzivi rows were already captured by their active ingredient, and no Jobevne rows were present. The remaining unmatched candidate names specified another ingredient or a device; these explicit ingredients were not overridden by a conflicting name or combination-regimen description. Aggregate checks are supplied in `drug_name_coverage_QA.json` and `name_audit_decision.json`.

Identity and ROLE_COD are retained from the **same DRUG row**, using the source quarter, report identifier and DRUG_SEQ. The definitions are: **any role**, any matching row; **PS+SS**, a matching row coded primary or secondary suspect; and **PS**, a matching row coded primary suspect. Any-role inclusion also retains other/unknown role codes if present. PS or SS status assigned to another medicine in the report cannot confer suspect status on the index agent. Repeated matching rows collapse to a single agent/report flag using logical OR.

Events are matched by case-insensitive equality to the complete trimmed REAC PT text. Repeated occurrences of the same term collapse to one report-level event flag. Reports may contain several agents and events, but each report contributes at most once to a given agent–event combination. No clinical adjudication of the recorded event or its culprit drug is inferred.

Within each version policy, the reporting universe contains all eligible reports, including those without a study agent. Its total and event margins are fixed across role definitions. The nonexposed row comprises reports without the index agent **in the specified role**, including index-agent mentions that occur only in excluded roles. Thus role restriction changes the exposure definition, not the common reporting background; it does not establish monotherapy or eliminate co-medication and indication confounding.

S6.3 Case-version selection and report flow

The early AERS fields CASE and ISR are mapped to case and report identifiers; later files use CASEID and PRIMARYID. Identifiers are kept as strings, and all drug/reaction joins use the same source quarter and report identifier. Within-quarter repeated DEMO keys are checked for contradictory CASEIDs and collapsed deterministically. Repeated snapshots of the same CASEID/report identifier across quarters retain the most recent source-quarter snapshot. Official deleted-case identifiers available through the last included quarter are excluded from both policies.

The **all-versions** policy retains distinct report versions after these steps. The **case-deduplicated** policy selects the latest complete FDA_DT within CASEID, breaking ties by the largest numeric report identifier and then the latest source quarter; CASEVERSION supplies a final deterministic tie-break. The selected record differs from the maximum available CASEVERSION in **104 CASEIDs**, reflecting date/version-order discrepancies. The specified date-first rule is retained and is not described as simply selecting maximum CASEVERSION or as an independently established FDA recommendation.

Selection precedes eligibility: a selected latest version must have a complete valid FDA_DT in the interval and at least one nonempty reaction in its matching source-quarter REAC file. An older eligible version does not replace an ineligible selected version. Administrative CASEID selection removes follow-up versions within the same case identifier; it does not match possible duplicate clinical episodes across different CASEIDs or establish unique patients.

The 21,911,439 source DEMO rows contained no missing case/report keys, within-quarter duplicate keys or contradictory case identifiers. Excluding 130,166 rows belonging to deleted cases and 891 repeated cross-quarter snapshots left 21,780,382 distinct report versions. Selecting one version per CASEID removed 3,254,559 additional versions before eligibility.

Stage	All versions	Case deduplicated
After deletion/snapshot handling and the specified version policy	21,780,382	18,525,823
Excluded: missing, partial or invalid FDA_DT	0	0
Excluded: FDA_DT outside the interval	2,677	2,646
Excluded: qualifying date but no nonempty reaction	65	51
Eligible reporting universe	**21,777,640**	**18,523,126**
Distinct CASEIDs among eligible reports	18,523,128	18,523,126

The two-CASEID difference in the last row reflects selection-before-eligibility. Final eligible records decrease by 3,254,514 (14.94%), which differs from the pre-eligibility version-removal count. `flow_qc.json` supplies all stages, including legacy-format counts, and `source_quarter_coverage.csv` records nonempty DEMO/reaction/target-drug coverage for every quarter. The deletion lists and their hashes are documented in `deletion_manifest.json`.

S6.4 Statistical and sparse-result presentation rules

RORs and log-Wald 95% CIs use the four-cell definitions and the observed a≥3/lower CI>1 criterion in S4.2. A 0.5 correction is added to all four cells when at least one cell is zero and all table margins are positive. A zero whole margin produces no numerical estimate. This sensitivity evaluates RORs; it does not present new PRR/IC concordance estimates.

The **reader-facing tables** uniformly suppress RORs and CIs whenever the uncorrected joint count is below three, regardless of ratio direction or confidence bounds. All counts, planned rows and signal flags are retained. Of 1,428 rows, **1,095 report an estimate, 249 have positive margins but a<3 and are labelled sparse, and 84 have a zero event margin and are not estimable**. All 84 zero-margin rows correspond to the original unmatched "bullous pemphigoid" string. Sparse and zero-margin conditions are distinguished in `estimate_display_status`, `arithmetic_estimable` and `ROR_reported`.

`role_dedup_presentation.csv` and `scar_role_presentation.csv` are the interpretation tables. `role_dedup_results.csv` and `scar_role_sensitivity.csv` retain arithmetic diagnostics, including continuity-corrected positive-margin estimates at a<3, solely for reproducibility. The latter values are not positive signals and should not be interpreted as increased risk. The rule changes display rather than the calculations, observations or signal criteria; `presentation_rule.json` specifies it.

S6.5 Role- and version-dependent findings

Version policy	Drug role	Signals: original 16 strings	Signals: separate PEMPHIGOID
All versions	Any role	119	9
All versions	PS+SS	117	9
All versions	PS	86	7
Case deduplicated	Any role	119	8
Case deduplicated	PS+SS	117	8
Case deduplicated	PS	84	7

There are **18 signal-classification changes among 714 paired role/agent/event comparisons** after CASEID selection: seven under any-role, five under PS+SS and six under PS-only. Equal signal totals do not imply unchanged individual pairs. `dedup_signal_changes.csv` gives every changed comparison with both sets of counts and CIs. Examples include loss of nivolumab–pruritus under any-role and PS+SS definitions, and loss of cabozantinib–PEMPHIGOID under the same definitions.

Several prominent patterns persist, with agent/event-specific exceptions. In the case-deduplicated PS-only analysis, vitiligo and maculopapular-rash signals remain for atezolizumab, pembrolizumab, nivolumab and ipilimumab. Durvalumab–vitiligo has only two reports, and tremelimumab–vitiligo has none; durvalumab–maculopapular rash has 19 reports and ROR 1.12 (95% CI 0.72–1.76), while the tremelimumab comparison has one report. These comparisons do not meet the signal criterion. PEMPHIGOID remains a signal for durvalumab, with 23 reports and ROR 3.74 (2.48–5.64), but the tremelimumab comparison has only one report. Tislelizumab and lenvatinib also retain PEMPHIGOID signals, precluding an ICI-

exclusive interpretation. All five anti-angiogenic/multikinase agents retain hand–foot signals under PS-only restriction, although several ICI comparisons also signal.

SCAR findings are likewise sensitive to the specified event and role:

Exact event; case-deduplicated analysis	Any-role signals	PS+SS signals	PS-only signals
Stevens–Johnson syndrome	10	10	9
Toxic epidermal necrolysis	10	8	5
Drug reaction with eosinophilia and systemic symptoms	3	3	0

PS-only SJS signals remain for the six more extensively represented ICIs and lenvatinib, sorafenib and regorafenib; the tremelimumab SJS count is four. TEN remains a signal for atezolizumab, pembrolizumab, nivolumab, durvalumab and ipilimumab, while tremelimumab has one report. No agent meets the DRESS signal criterion under PS-only restriction. These reporting results neither exclude clinically important reactions nor validate a drug-specific diagnosis; the clinical SCAR assessment framework draws on external clinical evidence. Complete SCAR results include negative and sparse comparisons in the files identified above.

S6.6 Files, software and verification

In addition to the tables and QC files cited above, `role_dedup/` supplies:

- `run_role_dedup.py`, `drug_dictionary.csv`, `event_dictionary.csv`, `protocol_locked.json` and `official_sources.md`: source-processing code, definitions and source documentation.
- `raw_source_manifest.json`, `deletion_manifest.json`, `source_quarter_coverage.csv` and `flow_qc.json`: source provenance, complete-quarter coverage and count flow.
- `reproduce_from_counts.py`, `make_presentation_tables.py` and `presentation_rule.json`: independent aggregate recalculation and deterministic display rules.
- `README.md` and `offline_reproduction_QA.json`: package instructions and the successful independent counts/presentation reproduction record.
- `independent_numerical_QA.json`, `independent_source_check.py` and `independent_source_check.json`: verification of all saved estimates and separate Python parsing checks of 2011Q1, 2012Q4 and 2026Q2 drug records. These representative source checks agree with the SQL-derived agent/role counts.
- `drug_name_coverage_QA.json` and `name_audit_decision.json`: aggregate name-ascertainment checks without individual report details.
- `requirements.txt`: source-analysis dependencies, tested with Python 3.12.10, DuckDB 1.4.3, pandas 3.0.2 and NumPy 2.3.5. The counts-only reproduction requires the Python standard library.

From the bundle root, run `python role_dedup/reproduce_from_counts.py --out-dir reproduced/role_dedup` to recompute all 1,428 diagnostic rows and verify their reader-facing

presentation without network access or report-level data. It checks the full comparison grid, nonnegative cells, margins, signal criteria, role nesting, fixed reporting background and case-selected counts. Full source rebuilding is separate: python role_dedup/run_role_dedup.py --source-root <extracted-official-quarterly-files> --out-dir <new-analysis-directory>; --source-root can be supplied more than once. Official source archives are identified by URL and checksum rather than redistributed as a report-level working database.

Independent arithmetic checks reproduce all 1,344 positive-margin RORs and both confidence limits to floating-point precision, confirm all 84 zero-margin rows, and reconcile the full flow. This verifies calculation and implementation consistency; interpretation remains subject to spontaneous-reporting, indication, drug-name ascertainment and residual cross-CASEID duplication limitations.
