# Supplementary material for: Cutaneous reporting patterns of drugs relevant to hepatocellular carcinoma: a cross-indication FAERS analysis and proposed management framework
Source: Front Med (Lausanne). 2026 Oct 30;13:1959163. doi: 10.3389/fmed.2026.1959163 (PMC13759389; doi:10.3389/fmed.2026.1959163)
Supplement: Supplementary file 3 [file Table1.docx]

# Supplementary Material — READUS-PV reporting checklist

Manuscript: *Cutaneous reporting patterns of drugs relevant to hepatocellular carcinoma: a cross-indication FAERS analysis and proposed management framework.*

Reported in accordance with the READUS-PV statement (Fusaroli M, Salvo F, Begaud B, et al. *Drug Saf* 2024; 47: 575–584). Section references are to the numbered sections of the manuscript.

## Main checklist

| Item | Checklist item (abbreviated) | Reported? | Location |
| --- | --- | --- | --- |
| 1a | Identify the study as a “disproportionality analysis”; specify data type and database(s) | Partially | Title identifies FAERS analysis; Abstract reports the disproportionality measures and database; keywords identify disproportionality analysis. |
| 1b | Report adverse event(s) and/or drug(s) under study | Yes | Title; Abstract; Methods §2.2 |
| 2a | Describe drug(s), utilisation, nature/frequency of event(s), existing knowledge | Yes | Introduction §1 (¶1–3) |
| 2b | Rationale for the analysis | Yes | Introduction §1 (final paragraph: cross-indication reporting patterns and partner-exclusion comparisons). |
| 2c | Why ICSR databases and disproportionality analysis are suitable | Yes | Methods §2.1 and §2.3 (spontaneous-report signal detection; no incidence or causality interpretation). |
| 3 | Specific objectives: event(s), drug(s), reference group, pre-specified hypothesis | Yes | Introduction §1; Methods §2.2–2.4 and §2.6; Supplementary Methods S1–S6 distinguish original objectives from added sensitivities. |
| 4a | Identify study type and data used (ICSRs) | Yes | Methods §2.1 and §2.6 |
| 4b | Outline of study design incl. primary and sensitivity analyses | Yes | Methods §2.3–2.4 and §2.6; Supplementary Methods S2–S6: ROR, PRR, IC, partner exclusion, phrase/exact matching and a separate quarterly drug-role/CASEID sensitivity. |
| 5a | Database name, custodian, coverage; drug/event coding thesaurus | Yes | Methods §2.1–2.2 and §2.6; Supplementary Methods S1–S3 and S6: FAERS/openFDA and official quarterly files, MedDRA-coded reaction text, original generic-name phrases and independently defined quarterly ingredient/trade-name matching. |
| 5b | Extraction dates; data pre-processing, transformation, exclusions | Partially | Methods §2.1–2.4 and §2.6; Supplementary Methods: July 2026 historical access month known; exact day and response metadata not retained. September 10 openFDA extraction uses first FDA receipt January 1, 2004–June 30, 2026. September 12 quarterly analysis uses 2011Q1–2026Q2 releases and FDA_DT January 1, 2011–June 30, 2026; source manifests and full preprocessing/flow are supplied. |
| 6a | Study population, including restrictions | Yes | Methods §2.1–2.2 and §2.6; Supplementary Methods S1–S3 and S6: cross-indication reports, 14 agents and 16 original phrases. Original analysis has no indication/date filter. OpenFDA and quarterly sensitivities use separate date predicates; quarterly analyses additionally specify same-drug-row PS, PS+SS, any-role and CASEID selection. |
| 6b | Nature and meaning of key variables | Yes | Methods §2.3 and §2.6; Supplementary Methods S3–S4 and S6 (ROR, PRR, IC025, PT strings, drug roles, FDA_DT and case/version identifiers). |
| 6c | Grouping of drugs/events; active ingredient vs trade name; selected role | Yes | Methods §2.2–2.4 and §2.6; Supplementary Methods S3 and S6: descriptive event groups, original harmonised generic-name phrases, complete-term matching and post-hoc PEMPHIGOID; fixed quarterly ingredient/trade-name definitions with same-row roles. |
| 6d | Additional data sources and how they interact with ICSRs | Yes | Methods §2.5 and Discussion: external guidelines and clinical studies inform the proposed framework; they are not record-linked to FAERS. |
| 7a | Descriptive analyses: variables, tests, thresholds | Yes | Methods §2.3; Results §3 (signal counts, cross-metric concordance) |
| 7b | Disproportionality measure(s) and signal threshold(s), with rationale | Yes | ROR: a≥3 and lower 95% CI>1; PRR: a≥3, PRR≥2 and Yates χ²≥4; IC: a≥3 and IC025>0 (Methods §2.3; Supplementary Methods S4.2–S4.3). |
| 7c | Sensitivity analyses / confounding control | Yes | Methods §2.3–2.4 and §2.6; Results §3.5–3.7; Supplementary Methods S4–S6 and complete tables. Partner exclusion and role/CASEID sensitivities are descriptive, with agent/event-specific exceptions; they do not eliminate indication or reporting confounding. |
| 7d | Variables/methods for case-by-case analysis; causality algorithm | Not performed | No clinical case-by-case adjudication. API version handling and independent quarterly CASEID-based latest-version selection are distinguished in S1/S6; matching different CASEIDs to clinical patients was not performed. ALDEN is an external SJS/TEN causality tool, not applied to these reports. |
| 7e | Statistical methods for other data sources | Not applicable | External clinical evidence is narratively synthesised; no new statistical pooling or linked-data analysis. |
| 8a | Number of ICSRs at each stage, with reasons for exclusion | Partially (historical); Yes (revision) | Historical background: 20,328,571 reports with a reaction; historical non-overlapping study-drug union not retained. September 10: 20,692,690 available minus 3 without reactions and 385 outside/missing the first-receipt interval =20,692,302; study-agent union 466,166. Quarterly sensitivity: 21,777,640 eligible report versions and 18,523,126 selected CASEIDs, with full exclusions in S6. Counts are reports/case identifiers, not verified unique patients. |
| 8b | Demographic/clinical characteristics of cases vs reference group | Not reported | Individual demographics and clinical case characteristics were not compared; quarterly administrative identifiers/dates served version selection, not clinical characterization. Comparability and confounding remain incompletely assessed. |
| 9 | All results with confidence intervals; results of sensitivity analyses | Yes, with historical limitations | All 18 selected Table 3 RORs have 95% CIs. Corrected historical primary/sensitivity presentation and complete revision main/partner/post-hoc estimates are supplied. All 1,428 quarterly comparisons, sparse display rules and signal changes are reported in Results §3.7 and Supplementary Methods S6. The original partner archive lacks sufficient margins for every CI; the new partner analysis supplies them. |
| 10 | Case-by-case analysis and causality assessment | Not applicable | Not performed |
| 11 | Discuss key results vs objectives and literature; expected vs emerging signals | Yes | Discussion — “Comparison with prior work” |
| 12a | External validity to the general population | Yes | Methods §2.1; Discussion limitations; Supplementary Methods S6: cross-indication reporting, source/drug-name differences and role-dependent sensitivity; no HCC-specific incidence or causality inference. |
| 12b | Potential relevance in clinical practice | Yes | Discussion; Tables 1–2; Figure 1. The proposed clinical framework uses external evidence and is not prospectively validated. |
| 12c | Propose further study designs | Yes | Discussion — call for a prospective, de-identified HCC × skin registry with external validation |
| 13 | General limitations (no causation/incidence) and specific limitations (confounding, reporting bias) and mitigation | Yes | Methods §2.1 (reporting ≠ incidence); Discussion — limitations paragraph |
| 14a | Source of funding/sponsorship and role of funders | Partially | Funding statement identifies the Jilin Province Tianhua Health Public Welfare Foundation, grant J2025JKJ012 to LY. The role of the funder is not separately reported. |
| 14b | Conflicts of interest | Yes | Conflict of interest statement |
| 14c | Institutional approval | Yes | Ethics statement: publicly available, de-identified FAERS data with aggregate results; no institutional approval was required for this analysis. |
| 14d | Data/code availability, software version, protocol registration | Yes (registration: none) | Data availability; Methods §2.3; Supplementary Methods and reproducibility bundle: software versions, definitions, counts, code and source hashes. The original analysis was not prospectively registered. The PEMPHIGOID correction and added quarterly sensitivity are identified separately; a specified analysis protocol does not imply prospective study registration. |

## Abstract checklist

| Item | Checklist item (abbreviated) | Reported? | Location |
| --- | --- | --- | --- |
| 1a | Aim/rationale for the study | Yes | Abstract Background and Methods: distinguish cross-indication reporting patterns and examine co-reported medicines. |
| 1b | Adverse event(s) and/or drug(s) under study | Yes | Abstract: 16 reaction search phrases; nine ICIs and five anti-angiogenic/multikinase agents. |
| 1c | Specific population or setting | Yes | Abstract Methods and Conclusions explicitly state cross-indication reports, without restriction to HCC; HCC is the clinical application context. |
| 2a | Identify as disproportionality analysis; specify data type | Yes | Abstract Methods explicitly identifies a disproportionality analysis using FAERS/openFDA reports. |
| 2b | Database name and type of access | Yes | Abstract (FAERS via openFDA) |
| 2c | Timeframe and geographical region | Partially | Abstract names the US FDA database; access month and unrestricted original reporting dates are specified in Methods §2.1. Reports are not geographically restricted to the United States. |
| 2d | Disproportionality measure(s) and significance threshold(s) | Yes | Abstract (ROR; signal = ≥3 reports and lower limit >1) |
| 2e | Whether a case-by-case analysis is performed | Not applicable | No case-by-case analysis |
| 3 | Main findings with precision (95% CI), plus case-by-case summary | Partially | Abstract reports point RORs and three-metric concordance; 95% CIs are given in Results §3 and Table 3 |
| 4a | Key conclusions | Yes | Abstract Conclusions: no HCC-specific risk or individual drug-causality claims; proposed framework with external evidence. |
| 4b | Acknowledge disproportionality is hypothesis-generating/refinement | Yes | Abstract Conclusions explicitly exclude HCC-specific risks and individual causality; framework and monitoring remain unvalidated. |
| 4c | Implications and clinical relevance | Yes | Abstract Conclusions: attention to concomitant medicines and a proposed externally informed clinical framework. |
